# Visualization analysis of author collaborations in schizophrenia research

**DOI:** 10.1186/s12888-015-0407-z

**Published:** 2015-02-19

**Authors:** Ying Wu, Zhiguang Duan

**Affiliations:** School of Public Health, Shanxi Medical University, South Xinjian Road, Taiyuan, China

**Keywords:** Schizophrenia, Collaboration, CiteSpace III

## Abstract

**Background:**

Schizophrenia is a serious mental illness that levies a heavy medical toll and cost burden throughout the world. Scientific collaborations are necessary for progress in psychiatric research. However, there have been few publications on scientific collaborations in schizophrenia. The aim of this study was to investigate the extent of author collaborations in schizophrenia research.

**Methods:**

This study used 58,107 records on schizophrenia from 2003 to 2012 which were downloaded from Science Citation Index Expanded (SCI Expanded) via Web of Science. CiteSpace III, an information visualization and analysis software, was used to make a visual analysis.

**Results:**

Collaborative author networks within the field of schizophrenia were determined using published documents. We found that external author collaboration networks were more scattered while potential author collaboration networks were more compact. Results from hierarchical clustering analysis showed that the main collaborative field was genetic research in schizophrenia.

**Conclusion:**

Based on the results, authors belonging to different institutions and in different countries should be encouraged to collaborate in schizophrenia research. This will help researchers focus their studies on key issues, and allow each other to offer reasonable suggestions for making polices and providing scientific evidence to effectively diagnose, prevent, and cure schizophrenia.

## Background

Schizophrenia is a serious mental disorder with a lifetime prevalence rate of 1% in the general population worldwide. It is characterized by abnormal mental functions and disturbed behaviors, which characteristically appear as a series of clinical features, such as positive and negative symptoms, and disturbances in basic cognitive functions [1-6]. According to a recent survey of the World Health Organization, it has been estimated that mental disorder ranks the first in terms of disability adjusted life years (DALYs) and will surpass that of cardiovascular disease, respiratory disease, and malignant tumors [7-9]. Thus, there is an immediate need to prevent and treat schizophrenia. Given the enormous complexity of this disease, it is particularly important that specialists in psychiatry and neuroscience research, and scientists in the biomedical field in general, collaborate through resource sharing, exchange of ideas, knowledge dissemination, and information acquisition.

However, there have been very few studies on scientific collaborations in schizophrenia research. In this study we analyzed author collaboration relationships as a way to help researchers focus their studies on key issues in schizophrenia, in the hopes of helping them prevent and treat this disease. An analysis of this sort can help clinical and research departments select experts in the field of schizophrenia, thus allowing research groups to improve the efficiency of their research work and provide scientific evidence and guidance for making policy.

## Methods

The dates for this study were taken from Science Citation Index Expanded (SCI-Expanded) via the Web of Science. The dates contained all documents which have the word “schizophrenia” in the title, abstract, or keywords, from January 1st, 2003 to December 31st, 2012. These documents included reviews, meeting abstracts, and manuscripts. There were a total of 58,107 records, each of which contained a title, abstract, author names, institutions, sources, and key words.

Authors’ collaboration relationships were usually researched by using software such as SPSS and Pajek mainly through dimension-reduced simplified algorithms which were basically simple static first-generation InfoVis techniques constrained by traditional tools and methods and simple graphic processing and drawing. This study used the most recent citation analysis and visualization software called CiteSpace III (Edition 3.7), which is based on a JAVA application. CiteSpace was invented by Dr. Chaomei Chen (School of Information Science and Technology, Drexel University, Philadelphia, PA, USA). Dr. Chen is an international expert in the field of information visualization. With the advantage of solid background of theoretical mathematics, computer science and computer software and conversance with library literature information, he firstly introduced Pathfinder—a kind of algorithm that seeks the key path—into network analysis and developed the CiteSpace series of application software employing the second-generation InfoVis technique which are adapted to multivariate, time-sharing and dynamic complex network analysis. By combining InfoVis technique and scientometrics creatively, Chen pioneered the comprehensive academic and applied field of visualization with knowledge management to a new stage of decision-making marked by the assistance of domain mapping and knowledge visualization. The CiteSpace series of utility software developed by Chen has 10,000 users in more than 36 countries and has become a popular tool in scientometrics [10-12]. In CiteSpace III, we specified the range of years to be analyzed, the length of time slices within the time interval, and three sets of threshold levels for citation counts, co-citation counts, and co-citation coefficients. The specified thresholds were applied to the earliest, middle, and last time slice, and linear interpolated thresholds which were assigned to the remaining slices [13].

## Results

### Analysis of author external collaboration relationships

**Figures 1:**
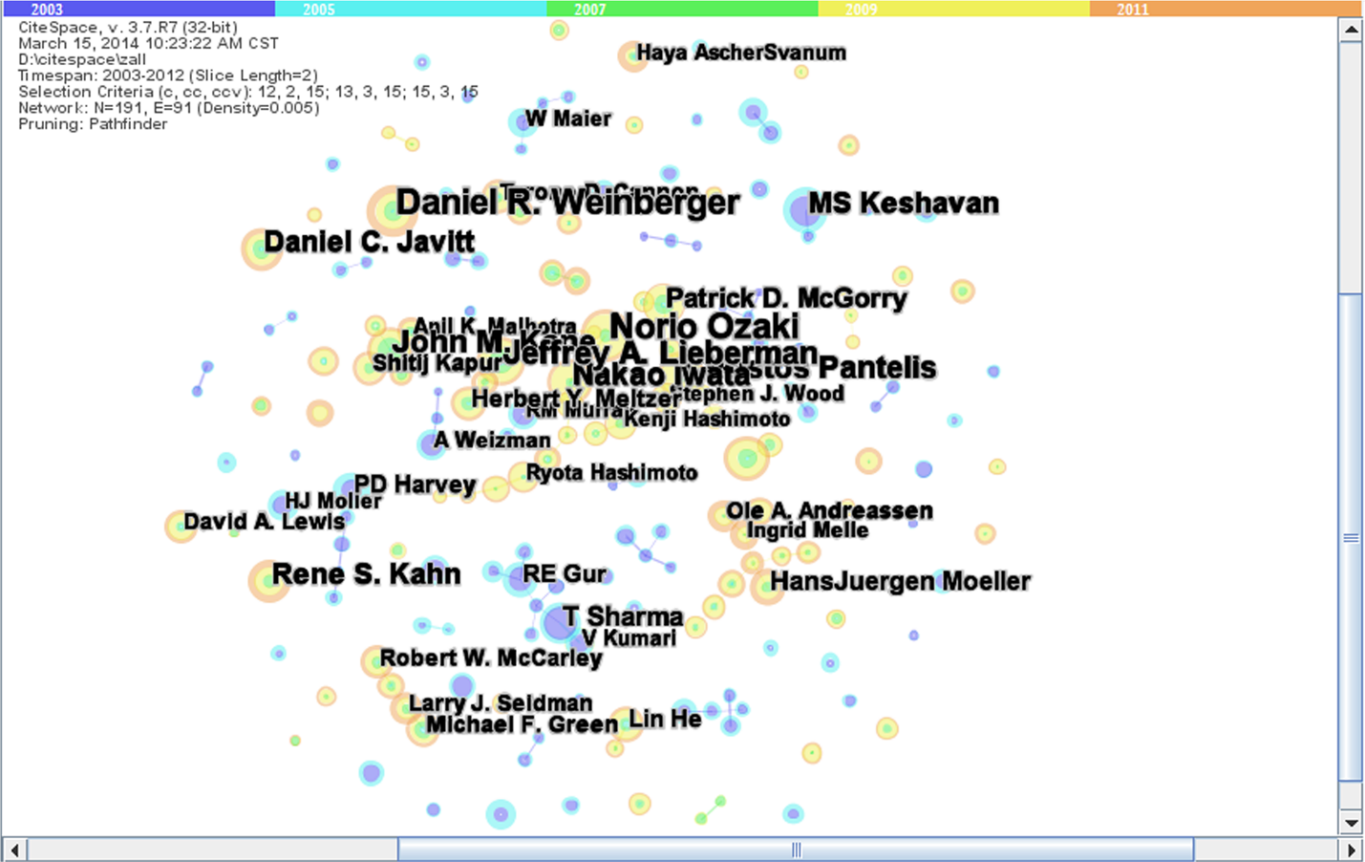
**Collaborative network of authors in schizophrenia field.**

Firstly, the datasets for the analysis of schizophrenia covering the time period from 2003 to 2012 were developed as a test bed for CiteSpace III. The lengths of time were sliced into five parts, each of which was 2 years. The three sets of threshold levels for citation counts, co-citation counts, and co-citation coefficients were (12, 2, 15), (13, 3, 15), and (15, 3, 15), respectively. Then, we selected an algorithm (Pathfinder) to determine the path into the network analysis. In the end, we chose corresponding nodes of networks and formed a citation network of authors in the field of schizophrenia (Figure 1). In this visualization, the node size represents the overall frequency of occurrence of authors, and the colored rings of the nodes represent yearly time-slices.

Table 1 lists the top 10 authors with regard to occurrence frequency in a collaborative network. For example, Norio Ozaki, Daniel R. Weinberger, Nokao Iwata, and MS Keshavan all focused on genetic research in schizophrenia. Norio Ozaki had the highest author occurrence frequency, and the paper with the highest citation frequency was one titled “Serotonin transporter missense mutation association with complex neuropsychiatric phenotype” published in 2003. The main research field of John M Kane and Daniel C. Javitt was drug treatment of schizophrenia. The highest citation frequency paper was titled “Tardive dyskinesia- prevalence and risk factor, 1959 to 1979” published in 1982 and “Recent advances in the phencyclidine model of schizophrenia” published in 1991, respectively. Jeffrey A. Lieberman’s field of research was nerve impulses in schizophrenia, and the paper with the highest citation frequency was one titled “Provocative tests with pycho-stimulant drugs in schizophrenia” published in 1987. The author Christos Pantelis conducted research in symptoms of schizophrenia, and the paper with the highest citation frequency was one titled “Neuroanatomical abnormalities before and after onset of psychosis: across-sectional and longitudinal MRI comparison” published in 2003. Robin M. Murray’s field of work was epidemiologic study on schizophrenia, and the highest citation frequency paper was titled “Is schizophrenia a neurodevelopmental disorder” published in 1987. Rene S. Kahn focused on neurochemistry of schizophrenia, and the highest citation frequency paper was “Metachlorophenylpiperazine as a probe of serotonin function” published in 1991.

**Table 1:** **Top 10 authors’ frequency in collaborative network**

Rank	Frequency	Author
1	70	Norio Ozaki
2	69	Daniel R. Weinberger
3	63	Jeffrey A. Lieberman
4	60	Christos Pantelis
5	60	Nakao Iwata
6	60	Robin M. Murray
7	59	John M. Kane
8	58	MS Keshavan
9	57	Rene S. Kahn
10	56	Daniel C. Javitt

**Figures 2:**
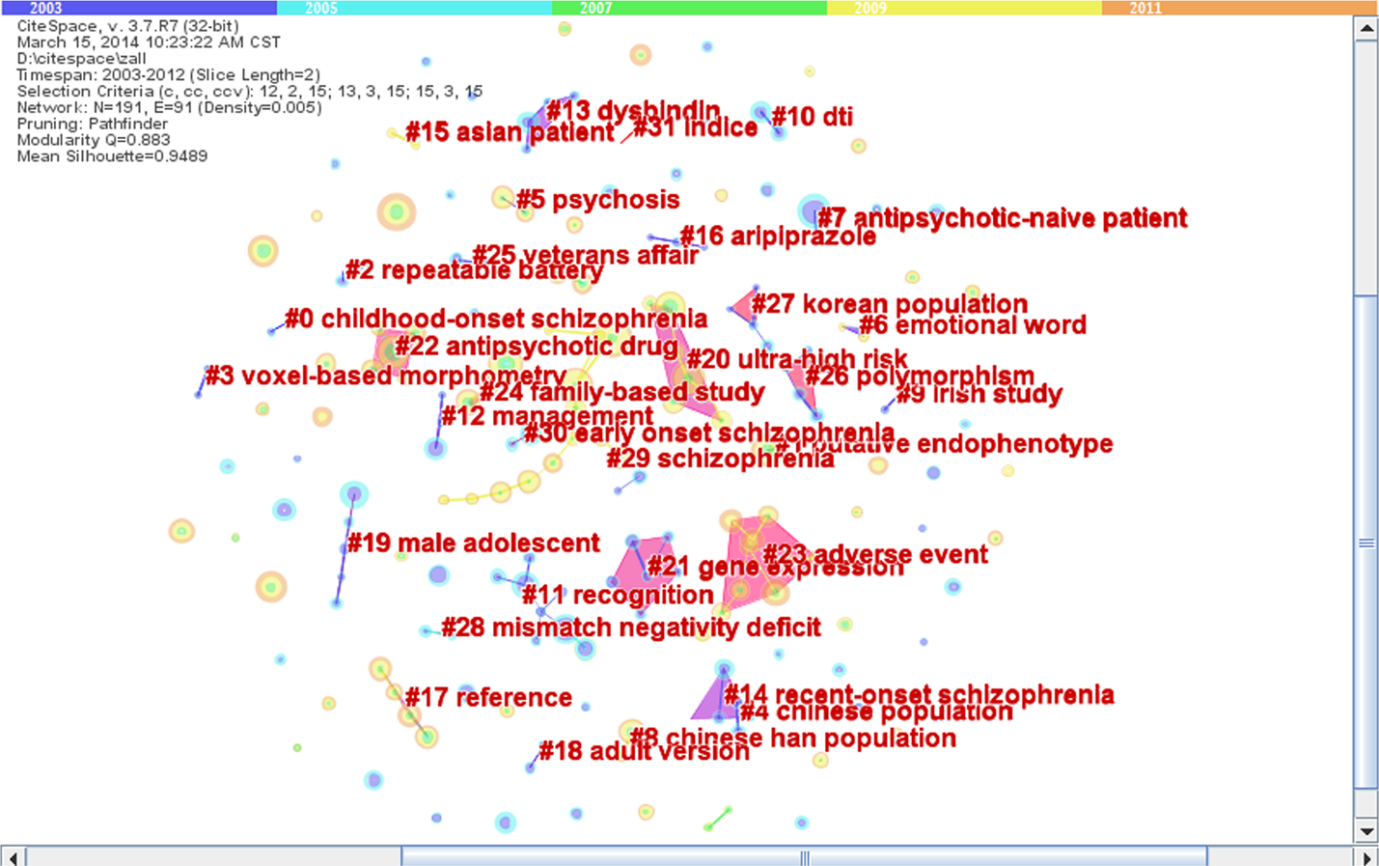
**External authors’ collaborative fields of different sub-networks.**

Hierarchical clustering analysis is widely used in analyses of collaborative networks. The authors who have frequency of occurrence which surpass the threshold are called core authors who have a large impact on similar authors. Through hierarchal clustering, we found 32 sub-networks. Figure 2 was a map of 32 clusters and the results were listed in Table 2. Sub-network 29 was the biggest sub-network, including 91 authors mainly concentrated on genetic research of schizophrenia. There were also seven sub-networks focused on genetic research of schizophrenia. Genetic research on the Chinese Han population was the most favored collaborative field in sub-networks 4 and 8, led by authors Lin He and GuoYing Feng, respectively. Sub-network 9 led by KS Kendler concentrated on schizophrenia genetic research of the Irish population. Sub-network 27 led by JJ Kim concentrated on genetic research of the Korean population. Sub-network 15 led by YuTao Xiang concentrated on genetic research of Southeast Asians. From these sub-networks, the regional features and genetic heterogeneity were most clear. Sub-network 13 and sub-network 26 focused on genetic research of schizophrenia as their main subject, led by authors W Maier and MJ Owen, respectively. Table 2 showed that sub-network 12 led by A Weizman, sub-network 16 led by R Marcus, sub-network 17 led by Larry J. Seidman, sub-network 22 led by John M Kane, and sub-network 24 led by Jeffry A. Lieberman, concentrated on drug treatment for schizophrenia. However, they never collaborated with each other on the topic. Similarly, sub-network 11 led by RE Gur, sub-network 14 led by MF Green, and sub-network 31 led by Philip D. Harvey focused on cognitive disorder in schizophrenia, and never collaborated with each other on the topic. The main field of sub-network 25, led by MT Tsuang, and sub-network 28 led by DL Braff was transmission of nerve impulse in schizophrenia. The topic of sub-network 3 led by EC Johntone and sub-network 10 led by RW McCarley was brain imaging in schizophrenia. The main collaborative field of sub-network 6, led by Cynthia Shannon Weickert, and sub-network 7 led by MS Keshavan was biochemical study on schizophrenia. Both sub–network 20 led by Christos Pantelis and sub-network 23 led by HanJuergen Moeller focused on symptoms of schizophrenia as their main subject. Sub-network 0 led by AW Toga and sub-network 19 led by PD Harvey concentrated on schizophrenia in childhood and adolescence. Sub-network 21 led by DR Weinberger and sub-network 30 led by S Frangon focused on neurochemistry in schizophrenia. Sub-network 1 led by Robin M. Murray centered on the epidemiology of schizophrenia. Sub-network 2 led by JM Gold concentrated on clinical assessment. Sub–network 5 led by Tyrone D. Cannon focused on etiology and pathogenesis of schizophrenia. The focus of sub-network 18 was substance-related study on schizophrenia and was led by J Klosterkotter.

**Table 2:** **External collaborative sub-network of authors**

Sub-network	Number of authors	Core author	Cluster top term	The main collaborative field
0	2	AW Toga	Childhood-onset schizophrenia	Study in childhood and adolescence
1	2	Robin M. Murray	Putative endophenotype	Epidemiologic study
2	2	JM Gold	Repeatable battery	Clinical assessment
3	2	EC Johnstone	Voxel-based morphometry	Etiology and pathogenesis
4	2	Lin He	Chinese population	Genetic research of Chinese Han population
5	2	Tyrone D. Cannon	Psychosis	Brain imaging
6	3	Cynthia Shannon Weickert	Emotional word	Biochemical study
7	2	MS Keshavan	Antipsychotic-native patient	Biochemical study
8	2	GouYing Feng	Chinese Han	Genetic research of Chinese Han population
9	2	KS Kendler	Irish study	Genetic research of Irishman
10	2	RW McCarley	DTI	Brain imaging
11	3	RE Gur	Recognition	Cognitive disorders
12	3	A Weizman	Management	Drug treatment
13	4	W Maier	Dysbindin	Gene research
14	3	MF Green	Recent-onset	Cognitive disorders
15	2	YuTao Xiang	Asian patient	Genetic research of Southeast Asian
16	3	R Marcus	Aripiprazole	Drug treatment
17	4	Larry J. Seidman	Schizophrenia	Drug treatment
18	2	J Klosterkotter	Adult version	Substance-related study
19	5	PD Harvey	Risk	Childhood schizophrenia disorders
20	7	Christos Pantelis	Taste	Psychopathology
21	7	DR Weinberger	Schizophrenia	Neurochemistry
22	5	John M. Kane	Antipsychotic drug	Drug treatment
23	10	HansJuergen Moeller	Adverse event	Psychopathology
24	3	Jeffrey A. Lieberman	Association study	Drug treatment
25	2	MT Tsuang	Schizophrenia	Transmission of nerve impulses
26	4	MJ Owen	Polymorphism	Genetic research
27	4	JJ Kim	Korean population	Genetic research of Korean
28	2	DL Braff	Mismatch	Transmission of nerve impulses
29	91	Norio Ozaki	Schizophrenia	Genetic research of Japanese
30	2	S Frangon	Early onset schizophrenia	Neurochemistry
31	2	Philip D. Harvey	Schizophrenia	Cognitive disorders

### Analysis of author potential collaboration relationships

**Figure 3:**
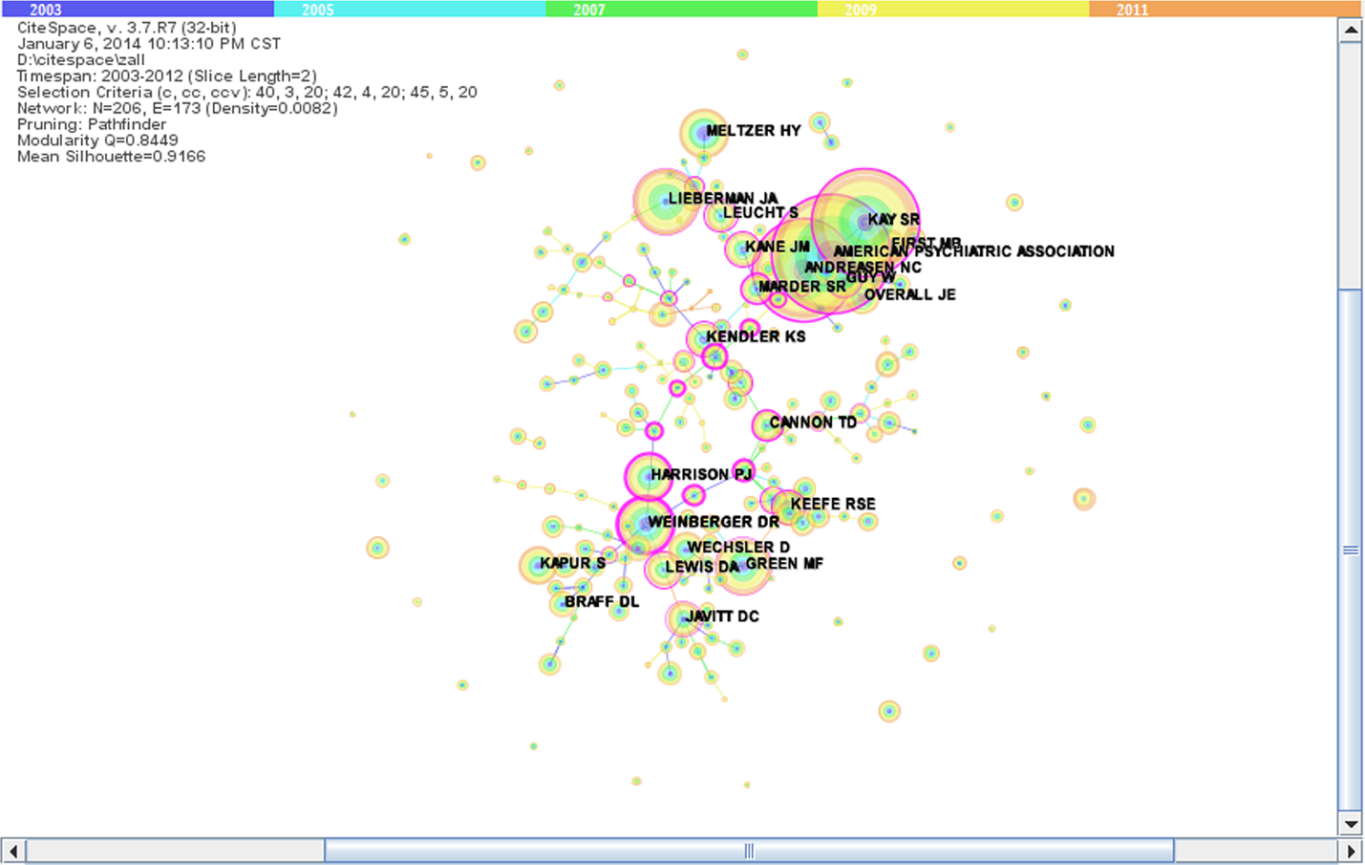
**Co-citation network of authors in schizophrenia field.**

We found potential collaboration relationships through co-citation analysis in the author collaborative network. The source of date was unchanged and the three sets of threshold levels for citation counts, co-citation counts, and co-citation coefficients were (40, 3, 20), (42, 4, 20), and (45, 5, 20), respectively. Then Pathfinder was used to determine the key path into the network analysis. We chose corresponding nodes of networks and formed a co-citation network of authors in the field of schizophrenia (Figure 3). In this manner, the authors who had the same research direction were connected together by author co-citation analysis; although in reality they may never have collaborated on a publication. The authors’ relationships has transformed from collaborating to publish a thesis into research direction.

We have listed the top 10 authors of co-citation frequency in Table 3. The highest co-citation frequency author was the American Psychiatric Association, which is the main professional organization for psychiatrists and trainee psychiatrists in the United States, and the largest psychiatry organization in the world. Its 36,000 members are mainly American, but some are also from other countries. The association has published various journals and pamphlets, as well as the Diagnostic and Statistical Manual of Mental Disorders, which codifies psychiatric conditions and is used worldwide as a key guide for diagnosing mental disorders. The highest citation frequency among its paper was “Reporting standards for research in psychology: Why do we need them? What might they be?” published in 2008. The second co-citation frequency author was Kay SR, who conducted research into the diagnosis of schizophrenia. The paper with the highest citation frequency among this author’s works was titled “The positive and negative syndrome scale (PANSS) for schizophrenia” published in 1987. This paper compiled the Positive and Negative Syndrome Scale. In 1990, Kay SR also introduced five of the axis symptoms of schizophrenia, including negative symptoms, positive symptoms, anxiety, depression, and cognitive problems. Since then, there has been closer attention on the cognitive symptoms of schizophrenia. The third co-citation frequency author was Andreasen NC who focused on the symptoms of schizophrenia. The title of the paper with the highest citation frequency for this author was “Family history method using diagnostic criteria-reliability and validity” published in 1977. The author Green MF focused on cognitive disorders of schizophrenia, and the title of the paper with the highest citation frequency was “What are the functional consequences of neurocognitive deficits in schizophrenia” published in 1996. Meltzer HY and Kapur S focused on drug treatment in schizophrenia. The highest citation frequency paper was titled “Classification of typical and atypical antipsychotic drugs on the basis of dopamine D-1, D-2 and serotonin 2 pki values” published in 1989 and “Psychosis as a state of aberrant salience: a framework linking biology, phenomenology, and pharmacology in schizophrenia” published in 2003, respectively. Harrison PJ concentrated on genetic research in schizophrenia and the highest citation frequency paper was “Schizophrenia genes, gene expression, and neuropathology: on the matter of their convergence” published in 2005. Wechsler D was a famous American psychologist and compiled the Wechsler Adult Intelligence Scale. The paper with the highest citation frequency for Wechsler D was “A standardized memory scale for clinical use” published in 1945. The authors Weinberger and Lieberman appeared in both Tables 1 and 3.

**Table 3:** **Top 10 authors’ frequency in co-citation network**

Rank	Frequency	Author
1	2341	American Psychiatric Association
2	2079	Kay SR
3	1970	Andreasen NC
4	1369	Lieberman JA
5	1137	Green MF
6	1073	Weinberger DR
7	1036	Meltzer HY
8	926	Harrison PJ
9	834	Wechsler D
10	822	Kapur S

**Figures 4:**
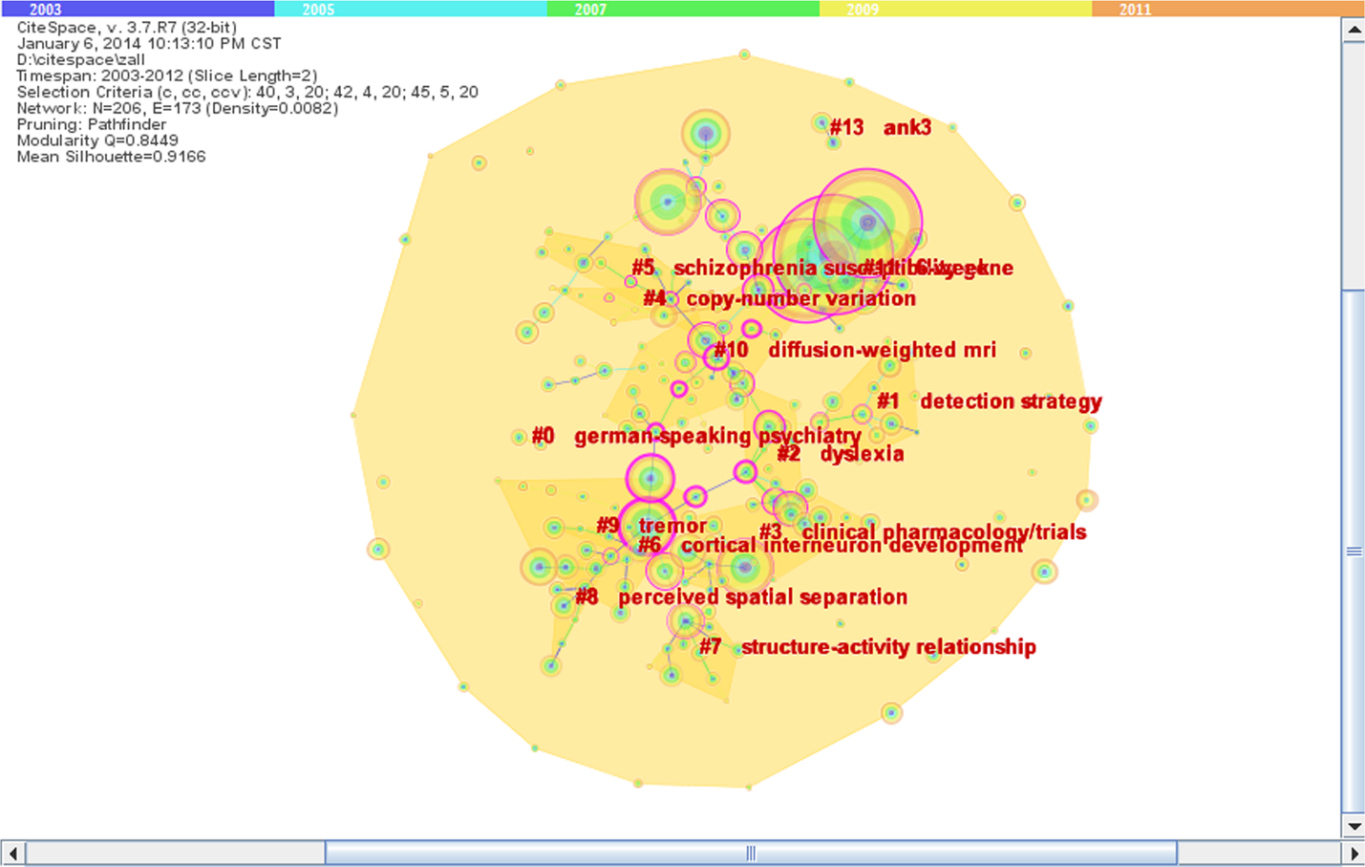
**Potential authors’ collaborative fields of different sub-networks.**

In order to further detect the potential author collaboration relationships, we found 12 sub-networks through hierarchal clustering. Figure 4 was a map of 14 clusters, and the results were listed in Table 4, which showed that sub-network 4 led by Stefanson H and sub-network 5 led by Kendler KS concentrated on genetic research on schizophrenia. Sub-network 2 led by Cannon TD, sub–network 3 led by Green MF, and sub-network 8 led by Braff DL all focused on cognitive disorders of schizophrenia as their main subject. Diagnosis of schizophrenia was the focus of research in sub-network 0 led by Kraepelin E and sub-network 1 led by Van Os J. Javitt DC and Weinberger DR led sub-network 7 and sub-network 9 to focus on neurochemistry of schizophrenia, respectively. Symptoms of schizophrenia was the main topic of sub-network 10 led by Andreasen NC and sub-network 13 led by Spitzer RL. As the central author in sub-network 6, Lewis DA concentrated on the etiology and pathology of schizophrenia. The American Psychiatric Association focused on the prognosis of schizophrenia in sub-network 11. The main research field of sub-network 12 was drug treatment for schizophrenia led by Lieberman JA.

**Table 4:** **Potential collaborative sub-network of authors**

Sub-network	Number of authors	Core author	Cluster top term	The main collaborative field
0	2	Kraepelin E	German-speaking psychiatry	Diagnostic standard
1	14	Van Os J	Detection strategy	Diagnostic standard
2	12	Cannon TD	Dyslexia	Cognitive disorder
3	18	Green MF	Clinical pharmacology/trials	Cognitive disorder
4	10	Stefansson H	Copy-number variation	Genetic research
5	11	Kendler KS	Schizophrenia susceptibility gene	Genetic research
6	8	Lewis DA	Cortical interneuron development	Etiology and pathology
7	12	Javitt DC	Structure activity relationship	Neurochemistry
8	8	Braff DL	Perceived spatial separation	Cognitive disorder
9	14	Weinberger DR	Tremor	Neurochemistry
10	19	Andreasen NC	Diffusion-weighted MRI	Brain damage symptoms
11	12	American Psychiatric Association	6-week	Prognosis
12	64	Lieberman JA	Nonfasting triglycerides	Drug treatment
13	2	Spitzer RL	ANK3	The positive and negative symptoms

## Discussion

In this study, we developed an author collaboration network for schizophrenia studies during dates ranging from 2003 to 2012 by using visualization technology. Our findings indicated that genetic research for schizophrenia was a very active and intense area of collaborative research. Schizophrenia is a complex mental disease [14], and genetics plays a key role in its pathogenicity, with a heritability factor of 0.70–0.85 [15-19]. Genetic inheritance of schizophrenia does not follow a Mendelian pattern. Genetic investigations focused on the elements of consanguinity, adoption, and monozygotic multiple births, and these investigations have shown that genetic inheritance is a major cause of schizophrenia. Thus, collaborative research has focused on finding susceptibility genes for schizophrenia. There is active research on finding the genes that correspond to the specific clinical symptoms of schizophrenia, which can help in providing personalized treatments based on one’s genotype [20,21].

In the authors’ external collaborative network, we found that the structure of the network was looser and there were fewer relationships among authors who belonged to different institutions in different countries. This indicated that collaborative networks typically tended to be within the same institution or in the same country or region. Because the level of scientific research is closely related with the rate of economic development, different countries have different scientific research levels. If the development of scientific collaborations among authors is confined to their own country, it is no surprise that polarization occurs, such that scientific research is higher in economically developed countries and lower in economically less-developed countries. This can prevent scientific research on schizophrenia to some extent. Thus, we should encourage collaboration among institutions and countries in order to promote coordinated development of international scientific research in schizophrenia.

In the authors’ potential collaborative network, we found that the structure of the network was more compact, which indicated that the research theme was relatively centralized and the collaborative relationship is stronger, allowing for collaborative publications focused on genetics, cognitive symptoms, and brain imaging for schizophrenia. However, researchers in the same field have a disincentive to collaborate and exchange knowledge because they are unable to know and communicate directly each other. Thus, the purpose of this study was to help with the exchange of ideas and strengthen communication between schizophrenia researchers to bolster research in this area.

The fact that Weinberger and Lieberman appeared in both the external collaboration network and potential collaboration network showed that these two researchers were not only prolific authors, but also published classical works which had great influence amongst their peers. They had made outstanding contributions in the field of schizophrenia research in the past 10 years.

## Conclusions

In recent years, schizophrenia research has become one of the most popular areas in the field of psychiatry. With the coming of biological information time, more and more researchers begin to find the necessity of scientific collaboration which not only is beneficial to sharing of ability and technology but also spreading of new knowledge, ability and technology and the production of new ideas, especially improved research production and influence of researchers. In this study we chose visualization technology to analyze author collaboration relationships in the field of schizophrenia so that leaders in the field can produce effective research groups, improve the efficiency of research work, and provide a basis for making policy. Furthermore, our findings may help in developing strategies for finding the etiology and pathogenesis of schizophrenia and help with clinical diagnosis and treatment.

Future studies from our groups will use visualization technology to track other branches in schizophrenia research, such as genome association studies, in order to acquire more valuable information and promote rapid development of this area.
